# Crafting Colonial Companions: Zionist Botany in Palestine, 1900‐1930s**

**DOI:** 10.1002/bewi.70036

**Published:** 2026-08-21

**Authors:** Mona Bieling

**Affiliations:** ^1^ History of Knowledge of Modern Societies Helmut Schmidt University Hamburg Germany

**Keywords:** Aaron Aaronsohn, botanical gardens, botany, colonialism, companion species, Otto Warburg, Zionism

## Abstract

In the early twentieth century, agricultural research played an important role in the Zionist movement's efforts to increase Palestine's productivity and to facilitate large‐scale Jewish settlement. Recent historiography has started to recognize the importance of agronomists and botanists as central actors in the broader project of Jewish‐Zionist knowledge production in and of Palestine. This article uses Haraway's concept of companion species to analyze two initiatives by Jewish plant researchers who, through different strategies, pursued a common goal: expanding botanical knowledge of Palestine, mobilizing plant life to advance political claims and alliances, and thereby contributing to the realization of a Jewish state. The case studies focus on the establishment of the first Jewish agricultural experiment station by agronomist Aaron Aaronsohn in 1910, and the creation of the botanical garden at the Hebrew University of Jerusalem by botanists Otto Warburg and Alexander Eig in 1931. Both projects exemplify how nature and culture were co‐produced in the service of Zionist nation‐building—using the Palestinian plant world to establish scientific authority, forge international alliances, and help delineate both epistemic and territorial boundaries.

## 1 Introduction

In recent years, the interplay between the environment and colonialism has received a lot of attention in environmental history, the history of science and adjacent disciplines. Since the foundational studies by Crosby and Cronon, the field has grown considerably.^1^ This article sees itself in conversation with this scholarship, especially works on colonial environmental knowledge and its networks,^2^ the importance of “useful” or “economic” plants for colonial projects,^3^ the specificities of settler communities and the environment,^4^ and the literature on the role of botanical gardens in this endeavor.^5^

For the history of Palestine/Israel, the links between the environment and Zionism have received attention since the 1990s with a focus on forestry and the role of trees as political tools for, among other things, settlement, land demarcation, and ideological attachment to the Jewish state.^6^ Recently, the research on the links between environment and pre‐state Zionism have been diversified by studies focusing on malaria and swamp drainage,^7^ Zionist climate research and responses,^8^ connections between religion, science, and animal bodies^9^ and urban settlers and their meat‐eating practices.^10^ Plants have not figured prominently in this literature, except for the above‐mentioned focus on trees and studies on the main export business of Mandatory Palestine, the orange industry.^11^

However, the historiography has started to recognize the work of agronomists and botanists specifically as a crucial contribution within the wider context of Jewish‐Zionist knowledge production of Palestine in the early twentieth century.^12^ It is widely acknowledged that agriculture played a central role within the early twentieth century Zionist movement.^13^ More specifically, agricultural research was crucial to increase Palestine's productivity in order to enable increased Jewish settlement. One group of practical Zionists, focusing on research to find the most effective plants to grow in this foreign‐to‐them environment of Palestine even acquired the label of “Botanical Zionists.”^14^ Used dismissively by their early twentieth century peers who worked in Zionism's main political and financial arenas, the term has recently been reclaimed by historians of the environment and of science to better understand the impact that plant research had on Zionist ideology and its practical application.

This article explores two examples that show how naturecultures can work to further a political project, in this case the Zionist goal of establishing a Jewish state in Palestine. *Natureculture* is a concept developed by Donna Haraway in her 2003 *Companion Species Manifesto*. Here, she lays out ways in which to overcome the divide between nature and culture and, instead, shows how to think of history as multispecies entanglements in which neither nature nor culture, neither the human nor the non‐human, could or should be neatly separated from each other.^15^ Of particular interest for the present analysis of Zionist plant research in Palestine is Haraway's concept of “companion species.” At the most basic level of understanding, companion species “make life for humans what it is—and vice versa.”^16^ Companion species are “bonded in significant otherness,” shaping life for each other by interacting over significant time spans.^17^ In Haraway's example, the companion species in question is her dog Cayenne; however, Haraway emphasizes that the concept is not limited to animals but that it includes plants and bacteria as well.^18^ Various scholars have taken up Haraway's concept, thinking through, for example, elephants, mushrooms, or microbiomes as companion species.^19^

This article discusses Zionist plant research in Palestine through the lens of companion species. It focuses on two attempts by Zionist plant researchers to create botanical companion species for their ideological goal of establishing a Jewish state in Palestine. I argue that the plant researchers in question realized the potential of the natural world, more specifically plants, for supporting their efforts at settling the land and making knowledge claims over it, two aspects which would aid the creation of a Jewish state in the practical and political realm. The two companion species in question are Palestinian wheat (*Triticum dicoccum dicoccoides*) and the botanical garden at the Hebrew University Jerusalem. In both cases, I am asking in which ways the interactions between the human (Zionist plant researchers) and the non‐human (wheat and garden) have shaped the humans and plants involved. I conclude that, while not an unalloyed success, in both instances plants played an important role in the ultimate realization of Zionism's goals in Palestine.

In the first instance of natureculture discussed here, the connections between Jewish agronomist Aaron Aaronsohn and a specific Palestinian wheat species helped forge ties with U.S. researchers and—crucially—U.S. funding for plant research in Ottoman Palestine. Palestinian wheat led Aaronsohn to connect Zionist plant research in Palestine with the American expansion towards its West and to a fraternization of the Zionist project with U.S. dryland research and agriculture for settlement purposes. As a result, the first Jewish‐Zionist agricultural experiment station in Ottoman Palestine, funded by American donors, was established in Atlit along the Mediterranean coast in 1910. This research station was the inception of institutionalized plant research by Jewish actors in Palestine.

Twenty years after this first experiment station in Atlit and after having built up several other institutions for plant research, Zionist plant researchers founded the first botanical garden in Mandatory Palestine. This botanical garden can be seen as the culmination of the institutionalization effort of plant research initiated by Aaronsohn. Both projects were loosely linked as Aaronsohn's experiment station in Atlit was a conceptual and institutional precursor for the department of botany at Hebrew University. The botanical garden was established in 1931 for the scientific study of Palestine's flora and had additional ideological‐political functions in support of the Zionist movement. In this instance, natureculture enabled and simultaneously challenged the claim for scientific self‐sufficiency that the Jewish community of botanists at Hebrew University Jerusalem tried to uphold through their garden. While consisting of many different plant species, botanical gardens achieve their signaling functions only by being seen and conceptualized as one entity.^20^ I am therefore referring to the garden as one companion species, emphasizing its unity over the plant diversity it consisted of.

For both companion species, I follow a broad understanding of how wheat and botanical gardens have impacted human history, summarized by Anna Tsing. Wheat and other cereals, in this understanding, have “domesticated humans” by settling them in agricultural societies.^21^ Botanical gardens, on the other hand, enabled colonial plantation landscapes by collecting, acclimatizing, and distributing plants for plantation systems—which, in turn, “were the engine of European expansion.”^22^ The article thus focuses on an in‐depth analysis of two specific examples of companion species in early twentieth‐century Palestine. As argued elsewhere in this special issue, small‐scale, time and place sensitive analyses of human/non‐human relationships are an important part of understanding the various shapes that naturecultures can take and express themselves in.^23^ This approach also helps understand the possible entanglements between nature, culture and politics and is needed in order to explore these questions in contested (colonial, imperial, national) spaces.

In both instances, Jewish‐Zionist scientists engaged with various colonial influences, emancipating themselves from the two colonial projects that ruled Palestine (the Ottomans until 1917 and the British from 1917–1948) while forging various alliances with others that seemed more useful to their state‐building project, as well as engaging in their own boundary‐making efforts. The article teases out the meaning of various colonial connections in early Zionist plant research in Palestine by applying the concept of colonial botany as described by Londa Schiebinger and Claudia Swan as “the study, naming, cultivation, and marketing of plants in colonial contexts.”^24^ Originally developed for a set of practices and material cultures in the early modern world, colonial botany has been applied to more modern contexts as well.^25^ It is a useful framework for reading the history of plant research in Palestine as it exposes human power relations in the process of knowledge creation, as well as the various scientific, political, and ideological intentions behind botanical practices.

## 2 Wheat as a Companion in the Forging of International Connections (1900–1914)

The first instance explores the entanglement between botanist Aaron Aaronsohn and a species of Palestinian wheat (*Triticum dicoccum dicoccoides*), which he used to forge ties with American researchers and donors. This collaboration enabled Aaronsohn to establish the first Jewish‐run agricultural experiment station in Ottoman Palestine. The wheat's specific botanical characteristics, together with colonial anxieties about the productivity of drylands in both America and Palestine, made such cooperation attractive. By positioning both himself and Palestinian wheat as native solutions to a global agricultural problem, Aaronsohn enhanced the appeal and credibility of his claims. By proposing Palestinian wheat as solution for successful Jewish settlement in Palestine and for American food security, I suggest that Aaronsohn was trying to fashion a companion species out of this plant due to its specific biological properties and its nativeness to the land Zionists claimed for themselves.

As part of his work involving plant research, Aaronsohn traveled widely throughout Ottoman Palestine and its neighboring regions, which made him intimately familiar with the land and its human and non‐human inhabitants. These qualities, mobility and great familiarity with the region, also made him a unique asset for the British war effort during WWI when he became the founder of a Jewish spy ring.^26^ However, more recently, historiography has reclaimed Aaronsohn's work as agronomist before WWI as similarly important for Zionist history as his political involvement during the war.^27^ This literature shows that Aaronsohn's botanical work cannot be divided from his political efforts. Two aspects of Aaronsohn's agronomic work are usually highlighted in this literature, one institutional and one scientific: First, his establishment and leadership of the first Jewish agricultural experiment station in Ottoman Palestine, founded in Atlit, south of Haifa along the Mediterranean coast in 1910. Second, his research agenda centering around *Triticum dicoccum dicoccoides*, which I am calling Palestinian wheat for reasons explained below, and his initial location of this species in Palestine.

Aaron Aaronsohn immigrated to Ottoman Palestine from Romania with his family in 1882 at the age of six. His parents were part of the so‐called First Aliyah, a group of 20,000–30,000 Jewish immigrants seeking to set up the first Jewish agricultural settlements, including the Aaronsohn's new home in Zichron Ya'akov. As a young man, Aaronsohn's great interest in the natural world caught the attention of French Baron Edmond de Rothschild, a wealthy supporter of Jewish settlement in Palestine, who sent him to the École Nationale d’Agriculture in Grignon, France, for his education in agronomy and botany. After two years, Aaronsohn returned to Palestine and held several posts as agricultural advisor to Rothschild and to the Ottoman administration. During this time, he established a reputation as a knowledgeable agronomist through publications, participation in scientific expeditions across Palestine, and collaborations on the practical applications of agricultural science.

A 1902 visit to Germany marked a turning point in Aaronsohn's career. Here, he met leading botanists Georg Schweinfurth and Otto Warburg, who encouraged him to return to Palestine in search of a specific wheat species believed to be the original wheat (*Urweizen*) from which all cultivated species developed. Identifying this alleged original species and proving its Palestinian origin would have important scientific and political consequences. In scientific circles, the *Urweizen* debate questioned the boundaries between botany and the humanities, as botanists sought greater authority in addressing fundamental questions of human development.^28^ Moreover, a lively debate was unfolding across various scientific circles in Central Europe over the location of the so‐called cradle of human culture. Locating the origin of human culture was deeply connected to locating the original uncultivated species of wheat, a staple in these ancient civilizations, and ultimately was supposed to answer the question where the biblical Paradise had been.^29^

Scholarship on Aaronsohn and Palestinian wheat puts different emphasis on the meaning of the interplay between the researcher and the plant. Historian Dana von Suffrin discusses how Aaronsohn's location of Palestinian wheat happened at a time when the role of natural sciences (here: botany) shifted and when it became possible to explain historical and cultural developments (such as the origins of civilization and agriculture) with botanical findings—with plants as proof for developments usually discussed within the humanities. According to her, Aaronsohn and the Botanical Zionists cleverly used the plant in order to play to the trends of the day and position themselves as inherently connected to the plant—and thus to legitimize their political endeavor.^30^ Historian Tobias Mörike reads the developments as “a key moment in connecting modern agriculture with biblical narratives.”^31^ Finally, Palestinian geographer Omer Tesdell argues that Aaronsohn appropriated a local plant through various scientific mechanisms and thus made Palestine attractive as a space for settlement. The scientific knowledge produced surrounding wheat was essential for making Palestine a space in need of improvement and thus implicitly a space for Jewish settlement.^32^ While von Suffrin and Mörike thus emphasize the connections between science and religion, between botany and cultural history, Tesdell emphasizes the colonial contexts of Aaronsohn's work.

During research trips in Palestine in 1906 and 1907, Aaronsohn located the species in question in northern areas of the country. The subsequent publications of his findings established wild wheat, *Urweizen*, or *Triticum dicoccum dicoccoides* more firmly within the scientific canon of the day.^33^ At the same time, this event and its subsequent scientific marketing catapulted Aaronsohn himself from scientific obscurity to the highest echelons of scientific and political circles in the U.S. From the outset, Aaronsohn was well aware of the scientific and political implications of his research agenda centered on locating and then experimenting with Palestinian wheat and he used them, as much as possible, to his and the wheat's advantage.

Aaronsohn's location of Palestinian wheat is an apt example for the linguistic challenges and epistemological consequences of a multispecies approach in contested spaces, especially in the context of colonial botany. The scholarship narrating Aaronsohn's location and collection of the plant describes this event either as a “discovery,”^34^ an “appropriation,”^35^ or a “rediscovery.”^36^ All of these terms carry a certain colonial meaning. The term discovery has a long colonial tradition and does not consider long‐standing local and indigenous knowledge and usage of a plant. Describing Aaronsohn's finding in these terms puts him into a long line of European plant hunters bringing back foreign species to European botanical centers.^37^ Describing Aaronsohn's actions as appropriation considers Palestinian local knowledge and the political context of settlement and extraction, in which these events played out.^38^ Lastly, the term rediscovery takes on a political layer of meaning in the context of Zionist settlement in Palestine. As some Zionists believed that the establishment of a Jewish sovereign state in Palestine was based on the land being the ancient homeland of the Jews and their immigration therefore a justified return, rediscovering the plant could refer to the knowledge of the Jewish inhabitants of the land in ancient times. Implicit in this view is the assumption that in between the first and second time Jews held knowledge about this plant, no significant development of knowledge or practices surrounding the plant existed. This assumption also fits into a widespread way of thinking by both European outsiders and Jewish settlers, which narrated the development of Palestine's environment as a story of thousands of years of degradation or, at best, stagnation.^39^

The second contentious aspect of the narrative of Palestinian wheat are the names that were given to this plant. After locating the species in question, and after confirmation of his findings by the group of German botanists who had set him on this research agenda initially, the species of wheat was declared to be the original wheat or *Urweizen* from which agriculture had developed in the distant past. Using the term original wheat or *Urweizen* solidified this line of thought and the scholarship behind it. Moreover, the plant has been described in the literature in two additional ways, namely as “wild wheat” or by its Latin name, *Triticum dicoccum dicoccoides*. Both terms, again, evoke colonial histories. Referring to the plant as “wild” erases any previous or contemporary Palestinian engagement with the plant. Presenting it as untouched and possibly even unknown (which, of course, stands in relation with the terms discovery and rediscovery discussed above) removes any knowledge and practices of local and indigenous people who had “cultivated” the wheat for “generations,” according to Tesdell.^40^

Lastly, the plants’ Latin name *Triticum dicoccum dicoccoides*, while being its approved scientific name, refers back to an even longer and more entrenched colonial past of European botany. Latin plant names are often seen as the most scientific or most objective ones when, in fact, they reflect the power that the Western scientific system of naming has held over the last three centuries. While a detailed explanation of how this so‐called binominal nomenclature was developed by botanist Carl Linnaeus and how it could gain precedence over other systems of knowledge is not within the scope of this article,^41^ it should be kept in mind that referring to the wheat on Aaronsohn's research agenda as *Triticum dicoccum dicoccoides* (as Aaronsohn himself did^42^) is conditioned by this longer history of Europeans’ scientific dominance.

In contrast to this, Tesdell has shown that Aaronsohn's classification practices of Palestinian wheat stood in tension with local Palestinian understandings of the plant in question. Tesdell quotes Aaronsohn's 1910 contribution to the *American Dry Farming Congress Bulletin* in which Aaronsohn reports sending local farmers out to collect Palestinian wheat (*Triticum dicoccum dicoccoides*). Much to Aaronsohn's bemusement, local collectors never brought back the “correct” specimen but provided him with a variety of barley which they called wild or devil's barley (“scha'ir barri” or “scha'ir iblisse,” *Hordeum spontaneum*). Upon Aaronsohn's request, however, these collectors were also agreeing with him that the specimens in question were wild wheat, “kamh barri.” Aaronsohn explained these interactions with a supposed cultural trait of politeness towards guests rather than well‐founded botanical knowledge of the local Palestinian farmers.^43^

In short, to understand the colonial implications of these plants and their study, it is necessary to unpack the terminology used to describe both Aaronsohn's act of locating the plant and the plant itself. After all, in this particular instance the colonial implications are manifold: they originate in a centuries‐old colonial history of European botany, to the erasure of local and indigenous knowledge and participation in this endeavour, but also to the specific and multilayered settler colonial context of Zionist politics in Palestine itself. Unpacking the terminology used for this plant also shows, following Spary's arguments about the unstable identity of botanical species, that the respective names for the wheat were always constructed categories.^44^ The remainder of this section will focus on this last aspect while referring to Aaronsohn's discovery as act of locating and to wild wheat as Palestinian wheat to try and avoid the pitfalls of reproducing colonial terminology.^45^

Successfully locating Palestinian wheat opened new possibilities for Aaronsohn's career as a reputable scientist and for his plans for establishing an agricultural experiment station in Palestine. The publication of Aaronsohn's findings by himself and Schweinfurth in several German‐language outlets attracted considerable attention outside of the Palestinian and German circles that had been involved up to this point, namely in the U.S. Department of Agriculture (USDA). Botanist David Fairchild, responsible for the introduction of new plants and seeds into American agriculture, invited Aaronsohn to the U.S. in 1909 in order to learn more about Aaronsohn's work and Palestinian wheat's agricultural potential.^46^ While Fairchild and other American scientists came to doubt the extent of the wheat's potential, their inclusion of Aaronsohn and his companion species into their research agendas showed the initial appeal of his claims and his success in enlisting this plant in the Zionist effort.^47^ On an individual level, Aaronsohn's connections with both German and American botanists were important for him and his research, yet they also played out within a context of long‐established international networks of scientific and plant exchange. After all, by its very nature, (colonial) botany was a practice that depended on cross‐border exchanges between and among people in the lab and the field, scientific centers and botanizing individuals, and across climate and vegetational zones.^48^

Palestinian wheat lent itself perfectly as a potential companion species for Zionist plant research as it could connect Palestine with dryland science in the U.S.: both places shared a similar climate (California being the model for U.S. dryland science), as well as anxieties about producing an adequate amount of food for its respective (future) populations. Desert anxieties were widespread in American scientific circles at the time, leading to U.S. experiments with alfalfa, dates, and camels, among other species.^49^ Moreover, both settlement projects were concerned about the relative lack of rainfall and the limited potential of intensive irrigated agriculture. Palestinian wheat and its constructed identity as being untouched by civilization further elicited the hope that some of its qualities, such as its resistance to heat, drought, wetness, frost and barren soils could be introduced into domesticated wheat species, thereby improving species’ climate and pest resistance and yield.^50^ To this end, American wheat research had already been ongoing for years when Palestinian wheat appeared on the scene as an additional species to experiment with.^51^ This entanglement unfolded at a crucial moment—the U.S.'s research agenda on wheat and its capacities for increased human settlement aligned well with Zionists’ research interests and needs of the emerging institutionalized plant research in Palestine.

Aaronsohn correctly defined the U.S. research agenda on dry farming as a possibility for his own work specifically and the development of Palestinian agriculture in general. During his trip around the U.S. in 1909, he spoke about his findings regarding Palestinian wheat and met influential American individuals active in agricultural and botanic circles. Fairchild, the head of the USDA's Section of Seed and Plant Introduction, helped Aaronsohn to connect himself in the U.S. He quickly became somewhat of a celebrity in the circles involved, even presenting his ideas to Theodore Roosevelt.^52^ Aaronsohn's goal in all of this was to build his reputation and present the “Auffindung” (locating, finding again)^53^ of and his research on Palestinian wheat so that U.S. funding for a Palestinian experiment station may materialize. In front of potential Jewish American donors and his general American audience, he argued that experiments with Palestinian wheat could lead to greater productivity of this species, which could, in turn, benefit U.S. (and global) agriculture as much as the Jewish one in Palestine. Writing for the Bulletin of the USDA's Bureau of Plant Industry, Aaronsohn summarized his findings regarding Palestinian wheat as follows:

The study of the wild types of our cereals should not serve merely botanical and historical ends. It has a practical, an economic—I may even say a social—importance. Its ultimate aim is to produce a little more bread at a little less expense where production is difficult and costly, and to render this production possible where up to the present time it has been impossible.^54^

By emphasizing the potential future productivity of Palestinian wheat, Aaronsohn played into contemporary American anxieties about food security and thus fell on sympathetic ears with his American audiences.^55^ Against this background, Tesdell sees Aaronsohn's usage of Palestinian wheat as deliberate in order to connect U.S. and Zionist anxieties about the productivity of the respective land on which their settlement efforts were based.^56^

While the USDA itself was not able to fund Aaronsohn's envisioned experiment station, he did manage to secure financial support of Jewish American philanthropist circles.^57^ In 1910, the experiment station at Atlit was finally opened, it being clear to everyone involved inside and outside of Palestine that it was an American station, based on American funding, chartered in New York, with a Jewish‐American board and with an American research agenda in mind.^58^ The experiment station focused mainly on two areas of research and experimentation, namely on Palestinian wheat as set out by Aaronsohn, and on Shamouti oranges, better known by their brand name Jaffa oranges.^59^ While Palestinian wheat held potential as an important source of nutrition, the orange industry was the most profitable agricultural undertaking at the time and held the promise to become a flagship export market for the Zionist movement.^60^

Moreover, an important factor in the attractiveness of Aaronsohn and his research agenda to U.S. officials was his claim to Palestinian nativeness of both himself and the plant he located. Aaronsohn's family was not native to the region, but Palestine was Aaronsohn's home, whose geography, flora, inhabitants, and languages he knew intimately. In some of the literature, Aaronsohn is described as indigenous or native, or as “a son of the land.”^61^ Keeping in mind the implications of linguistic usage described above, the term local might be better to refer to Aaronsohn and his knowledge of Palestine. This local knowledge was also what made him such a good candidate to be sent out by the German group of botanists to search for Palestinian wheat in the first place. The distinction here between local plant hunters and metropolitan scientific botanists is an interesting one, which again carries a long colonial history. As Shaul Katz has argued, Aaronsohn's findings in the field could only become officially acknowledged scientific knowledge after confirmation by the German botanists.^62^ As such, despite his Western (French) education, Aaronsohn stood in a long line of local plant collectors in the colonies whose findings were dependent on validation from European scientific centers. In this sense, his local standing on the one hand enabled him to apply very specific place‐bound knowledge but, on the other hand, excluded him from the community of Western scientific botanists and their scientific practices of publication and classification.

If his local standing excluded Aaronsohn to a certain extent from German scientific botany, it benefited him in his dealings with the U.S. officials who were looking for localized and specialized expertise for providing answers to their dry land farming obstacles. Tesdell even argues that Aaronsohn played up the nativeness of Palestinian wheat in front of his American audience and that,

Aaronsohn's wheat could then go to the United States as an isolated, self‐formed technical achievement, a valuable genetic resource rescued from an environment of Oriental backwardness, rather than as the product of a local plant‐human spatiality produced by the accumulated expertise of Palestinian cultivators.^63^

Performing nativeness could thus have different results in different contexts: in his dealings with the German botanists, Aaronsohn's local standing set him apart from science; and in his relationship with the U.S. officials, his claims to nativeness of himself and the plant supported his agenda around Palestinian wheat.

The first Jewish‐Zionist agricultural experiment station only operated for five years until the outbreak of WWI and Aaronsohn's and his colleagues’ shift from focusing on plant research to helping the British war effort. Both goals, however, were connected in their aim of helping to create the basic conditions for a viable Jewish state in Palestine, be it on the agricultural end or the political one. Despite its short institutional lifetime, the experiment station was important for plant research in Palestine. It was the first institutionalized effort at plant research by the Zionist community in Palestine. Moreover, it brought together Jewish individuals from various national and professional backgrounds who would continue working together to establish a scientific base and institutional framework for the study of Palestine's flora and its agricultural potential.

Palestine wheat continued to play a role, albeit marginal, in Zionist plant research. Aaronsohn and his sister Rivka carried out experiments with the plant at their station in Atlit. In 1918, geologist Max Blanckenhorn reported in a study on the soils of Palestine that research stations in various countries had achieved promising results, testing the plant for its resistance to various climatic conditions. Future experiments, it was hoped, would result in a highly productive and resistant plant with which one could “wrest a large piece of culture from the steppe and desert.”^64^ In the 1920s, crossbreeding experiments were carried out by the researchers at the successor institution to Aaronsohn's station in Atlit. Crossbreeding Palestinian wheat with imported wheat species was supposed to connect the desired qualities of the local variety with the good grain and baking quality of the imported varieties.^65^ In the 1920s and 1930s, botanist Alexander Eig, co‐founder of the botanical garden, also carried forth work on Palestinian wheat with his research on sweet grasses, the family under which Palestinian wheat was classified.^66^ Despite all this activity there was also criticism regarding Aaronsohn's portrayal of the wheat as a solution for both Zionist and American anxieties. Botanist and geneticist Nikolaj Vavilov considered Aaronsohn's claims as highly exaggerated from the start.^67^ Even Fairchild, Aaronsohn's loyal ally in the USDA, wrote in 1938 that “nothing spectacular” had resulted from the wheat research and that “the exaggerated claims made about it are strange reading now.”^68^ However, in 2015, the projection of almost endless productivity onto the wheat reared its head yet again when the Israeli company NRGene successfully decoded the full genome of this “national treasure” in order to “revolutionize cereal production and fight the world hunger crisis.”^69^ To what extent Zionism will affect Palestinian wheat in the future thus remains to be seen.

Although Palestinian wheat ultimately did not play a lasting role in either U.S. or Palestinian/Israeli agriculture, it helped lay the institutional structures for a longer trajectory of plant research in the region. In the context of the Atlit experiment station, colonialism was expressed through settlement and the creation of a new life in a foreign place. As a staple crop central to subsistence, wheat became a powerful symbol of this settler‐colonial vision—representing the potential to root a people in a new territory. In this instance, wheat as a companion species thus shaped the relations with foreign ideological and financial supporters and set the Jewish botanists in Palestine on their path for the institutionalization of their field.

## 3 Hebrew University's Botanical Garden: A Companion for Claiming Scientific Validity (1920s–1930s)

Since the demise of Aaronsohn's experiment station in Atlit during WWI, there had been several attempts at institutionalizing plant research in Palestine. Most notably, one of the aforementioned intellectual sponsors of Aaronsohn, German botanist Otto Warburg, was involved in several of these projects.^70^ Warburg, having held important positions within the Zionist movement from the 1900s onwards, was also one of the main drivers of plant research in Palestine, now under British Mandatory rule. In 1921, he was involved in the establishment of an agricultural experiment station close to Tel Aviv, led by agronomist Yitzhak Elazari Volcani, which was tasked with carrying out experiments and research in botany, agriculture, horticulture, and other areas that were supposed to contribute the improvement of agricultural practices in Palestine, with the goal to facilitate Jewish settlement.^71^

In 1925, Warburg invited botanist Alexander Eig to join him at this experiment station. In 1928, after a thorough restructuring of the experiment station and the integration of several of its research sections into the Hebrew University, the latter's Department of Botany came into existence with Warburg at its head.^72^ The establishment of the Hebrew University was a significant step towards Jewish self‐determination in Mandatory Palestine. The university had been founded in 1918 by the Zionist community in Palestine and inaugurated in 1925 with the first three research institutes being microbiology, chemistry, and Jewish studies. Throughout the 1920s and 1930s, the University was progressively built‐up while being under relative financial strain and often facing a lack of adequate teaching and research personnel for the envisioned faculties and departments.^73^

Eig and Hebrew University's botanical garden have received scant attention from historians compared to Aaronsohn, Warburg, and the earlier efforts involving plant research.^74^ In light of recent scholarship on the connections between politics and gardens, however, the botanical garden and its lesser‐known co‐founder deserve scholarly attention as the garden served important scientific and political goals. Similar to Aaronsohn, Eig can be regarded as a local in Palestine. He immigrated to Ottoman Palestine from Minsk with his family in 1908 at the age of fifteen. His research on the flora of Palestine contributed substantially to the botanical knowledge of the area. Eig died from cancer at only 44 years of age in 1938; even so, his work had profound impact on the development of Israeli botany through his colleagues who built on and engaged with his studies for years after his passing.^75^

There is ample scholarship to show the various ways in which botanical gardens are deeply entrenched in colonial history. They have been used by humans as research hubs for knowledge creation about economically useful plants, as acclimatization centers for such plants’ introduction into foreign climates, and as center for exchange of knowledge and plant materials between the different colonial projects. With the help of botanical gardens, European colonial powers have created colonial landscapes, often dominated by plantations, that catered to the colonizers’ goals of maximum agricultural productivity and efficiency.^76^ Botanical gardens were also used as “tools of empire”^77^ that sent out plant collectors and facilitated often illicit species extraction from non‐European climates. The most well‐known case of plant smuggling might be the illegal cinchona extraction from Peru and its subsequent significance for the European colonization of Africa.^78^

The botanical garden at Hebrew University Jerusalem served the Zionist movement in several ways. First, it was the fulfilment of their signaling ambitions to the outside scientific world, showing that an all‐Jewish enterprise was able to contribute in meaningful ways to Western scientific communities. Second, the garden made a knowledge claim over the space that should fall within the borders of their future Jewish state by including only the plants of Palestine. Third, the garden symbolized the Jewish scientists’ attempted emancipation from other colonial influences, especially the British, and their claim for self‐sufficiency—which could not always be obtained. As a result, in contrast to the Palestinian wheat, the botanical garden was a conscious effort to build a Jewish nation independent of outside support. I argue that in the case of the botanical garden, Eig and Warburg fashioned it as a companion species for Zionism with the goal of establishing the Jewish scientists at Hebrew University as experts over the knowledge of Palestine.

Starting in the late‐1920s, Warburg and Eig actively worked towards the establishment of a botanical garden on the premises of the Hebrew University Jerusalem. During those years, Warburg was the official head of the Department of Botany and Eig his deputy and custodian of the herbarium. At the time, the university was still in its infancy and funding, as well as land on which to establish a garden, were scarce. The process therefore entailed some intense lobbying and negotiating efforts by Warburg and Eig until the garden could be inaugurated in 1931 by Eig who planted the first tree on the premises.^79^

Warburg and Eig intended the garden to be a scientific tool for research and teaching, integrated into the Department of Botany. However, not all Botanical Zionists agreed with their approach to integrate botanical research at the university. For example, the head of the experiment station, Volcani, criticized the “scientification” of botanical and agricultural work by integrating these into the university. Volcani feared that the work at the university would encourage the increasing production of theoretical knowledge, rather than its practical application. He was concerned about a certain “city mentality” of the Jewish scientists and their lack of practical skills.^80^ In voicing these concerns, Volcani employed familiar tropes about the alleged diaspora mentality of the Jewish settlers in Palestine and the need to create a “New Jew” along with a new flora.^81^

One aspect that hindered the establishment of the garden was the lack of adequate land, which would also be conveniently located close to the university buildings on Mount Scopus. In the process of searching for said land, the importance that the involved botanists and university officials attached to the garden becomes clear. Warburg was willing to accept land that would, for botanical reasons, not have been entirely adequate for planting a garden, reasoning that any form of garden would be better than none at all.^82^ On the other hand, university chancellor Judah Magnes (who had also been one of the Jewish American philanthropists funding Aaronsohn's experiment station), seemed annoyed by the prospect that the 54 dunams of land suggested by Warburg and Eig as necessary for the garden would not materialize. In his negotiations with a British official, Magnes was told that the British government was not willing to expropriate the local Palestinians at this point, and that he could probably only buy 36 dunums of land for the garden, after careful convincing of the local inhabitants. Magnes retorted that “the University was interested primarily in the Botanical Garden and not in the Lifta Arabs.”^83^ Magnes was thus interested in securing the land, and not in lengthy negotiations about it.

The garden figured so prominently in the botanists’ minds because it was not only supposed to fulfill its main functions of researching and teaching botany in the university context. Its additional political functions and meaning become clear in a letter, written by Eig in July 1929, in which he discussed the creation of a botanical garden connected to the university, detailing questions of its layout, funding, and land acquisition.^84^ The letter shows that the garden was intended to do some heavy ideological lifting for the Zionist movement. In this letter, Eig delineates the Jerusalem botanical garden from other gardens in the region as being the first “true” botanical garden in the Middle East, rather than a mere “magnificent park” as in the case of the gardens in Alexandria, Cairo, and Beirut.^85^ By saying the Jerusalem garden was a “true” botanical garden, Eig alluded to the scientific principles by which the plants in the garden were arranged, “serv[ing] the science of Botany in all its aspects;” in contrast to the gardens of Egypt and Lebanon that, according to him, arranged their plants with ornamental functions in mind.^86^

This emphasis on the scientific direction of the garden was a recurring theme, which also played a role in the botanists’ ability to acquire funding.^87^ Emphasizing the scientific direction of the garden, and the botanic knowledge necessary to establish such a garden, served to support the reputation of the young Hebrew University and its all‐Jewish attempt at institutionalized knowledge creation. The garden was supposed to signal to the global scientific community that there was a Jewish scientific community present that could make knowledge claims over the space into which most of them had recently immigrated and where they were planning to build a state. Doing this in the field of botany, a field that was so inherently connected with the land, also signaled that this Jewish community knew the land well—a step towards gaining legitimacy for the Zionist project.

Both founders thus saw the garden as an important tool for the study of botany, but also as conducive to the prestige of the Jewish community in Mandatory Palestine as a scientifically independent and modern nation. This argument is in line with the connection between science and modernity and the claim that a modern state is a state with modern scientific institutions.^88^ However, in the case of Palestine, this scientific understanding of modernity and technologically driven projects always had to contend with religious ideals of the future of the Jewish nation as well. As historian Tamar Novick has shown, technology and religion worked together to create a modern Jewish state in Palestine.^89^ In the case of the botanical garden, its scientific direction was challenged by a competing scheme for a so‐called Garden of the Prophets and Sages, conceived by Ephraim Hareuveni, based on a religious concept of botany. The ultimate establishment of a scientific botanical garden, rather than a biblical one, was due to a combination of practical circumstances, personal characteristics of the botanists involved, and the political signaling functions attached to it.^90^

Ever since their creation, botanical gardens have combined the ordering of natural space with cultural, economic, medicinal, religious and other functions.^91^ Botanical gardens are curated spaces that include and exclude plants, depending on the intended function of the respective botanical collections.^92^ In the case of the Jerusalem botanical garden, the connection between the plants in the garden and knowledge about the land more broadly was made by its layout. It was not conceived as a typical twentieth‐century botanical garden, containing as many plant species as possible ordered by regions of the world. Rather, the Jerusalem garden combined practical and experimental sections with sections for illustrative purposes, focusing on Palestinian plants. According to Eig, the unique location of Jerusalem at the border of the Oriental and Mediterranean floral zones rendered the garden a special significance for botanical research.^93^ The focus was, therefore, on acquiring “a fuller representation of the Mediterranean and Oriental Flora” and to “concentrate in [the] garden nearly all Palestinian plants,” as well as “the most important of the neighboring countries.”^94^ At a time when the borders of a future Jewish state in Palestine were still unclear, gathering the plants of the area in question under one framework can be interpreted as a border‐making exercise. By combining all the plants of Palestine within one space and simultaneously making a knowledge claim about it, Eig and his colleagues solidified the space they saw as their homeland as a coherent (botanical) entity about which they could make scientifically sound statements.^95^ The garden was thus envisioned as a companion species for the Zionists’ scientific claims over Palestine.

In contrast to Palestinian wheat, the Jerusalem botanical garden was intended as a scientific and political project that would rely solely on Jewish‐Zionist expertise and would communicate Jewish independence and self‐sufficiency in Palestine. One aspect in which this becomes clear is Eig's claim that only the Jerusalem botanists could provide botanical institutions abroad with highly coveted plant specimen from “the holy lands,” namely Palestine, the Sinai, and Syria.^96^ Research on specimen exchange within the context of colonial botany has shown that the connections established by transnational and transimperial exchange of plant material could both foster connections and sustain dependencies.^97^ Therefore, establishing connections with institutions abroad, albeit in the seemingly unpolitical context of botanical exchange, embedded the Jewish botanists more firmly into an international network of scientists and created legitimacy for them individually as well as their institution.

However, Eig's claim that nobody was able to fulfil the demands from abroad regarding specimen from the area was exaggerated. The British Mandate forestry service in Palestine had a very active botanical section, which carried out research and sustained a lively exchange of plants, seeds, and other flora with countries abroad.^98^ The British thus maintained a global trading network of specimen that also ran through Mandatory Palestine. Moreover, British documents reveal that the Hebrew University's Department of Botany and the British forestry service's botanical section in Palestine did establish relations. In 1935, a British forestry report stated that “[l]iaison with the Botanical Department of the Hebrew University has been maintained by the exchange of visits and material and mutual assistance.”^99^ The forestry service, in turn, was in touch with Kew Gardens, sending specimens to London that needed further identification or that were duplicates to be added to Kew's collection. The British foresters in Palestine even stated that they were “much indebted” to Kew regarding the latter's support in scientific matters.^100^ If Eig and his colleagues were thus possibly not directly in exchange with Kew, they still operated within the British imperial space. Even when the Jerusalem botanical garden was supposed to communicate Jewish self‐sufficiency across borders, it was difficult to escape imperial ties in the realm of botany.

More fundamentally speaking, striving for self‐sufficiency in the realm of early twentieth century botany could only ever work on the discursive and symbolic levels. As most botanists at the time, Eig and his colleagues depended on international education, research and field trips across borders, cooperation with other (and more established) institutions etc. Eig and his colleagues traveled extensively through Europe and West Asia and relied on local help and international research exchange for their work.^101^ For example, Naomi Feinbrun‐Dotan, the only female scientist in the department, spent time in Berlin and Paris for research in the early 1903s and, between 1926 and 1944, conducted various research expeditions throughout Palestine as well as in Jordan, Iraqi Kurdistan, Lebanon, Sinai, Egypt, and Cyprus.^102^

Another way in which setting up the garden functioned as a boundary‐making effort was in relation to the Arab Palestinian plant knowledge existent in Palestine. While the garden incorporated mostly plants growing in Palestine, native Palestinian knowledge was not systematically incorporated into its set‐up. While some members of the Department of Botany spoke Arabic, they were not in close contact with native Palestinians and their knowledge about and uses of Palestinian plants. One exception was Ephraim Hareuveni, another Jewish botanist working at the Hebrew University. Hareuveni was an advocate for so‐called biblical botany and the creation of a garden that would recreate the landscapes of the Bible.^103^ On Warburg's initiative, Hareuveni had been working with the botanists at the Department of Botany for a while but found a more permanent employment at the Institute for Jewish Studies. As a rival for Warburg's and Eig's scientific botanical garden, the relationship between Hareuveni and the scientific botanists at the university was ambivalent.

Of the Jewish scientists discussed here, Hareuveni was the one most connected with native Palestinians and their plant knowledge, for example through a small museum in which he and his wife Hannah showcased their private Palestinian plant collection and offered classes and workshops to local students, teachers, and farmers. Several university officials mention that Hareuveni incorporated Palestinian plant knowledge into his work, which, however, came with certain qualifications.^104^ Hareuveni's botanical work and his inclusion of Palestinian expertise were categorized by himself and others as cultural rather than scientific knowledge.^105^ To gain local plant knowledge, he visited Arab villages where he thought to recognize plants from the Bible and Talmud, including their specific ways of cultivation. Studying Arab culture and observing local plant knowledge applied in the fields had the goal of learning more about the Holy Land's vegetation during Biblical times. Part of this “Arab ethnobotany,” as Mörike has called Hareuveni's approach, was the disregard for neophytes (non‐native plants), including Zionist agricultural crops.^106^ Therefore, Hareuveni did not necessarily value Palestinian expertise as such. Rather, he thought contemporary Palestinians to be vessels for the knowledge of the ancient Israelites whose expertise would be contained in and passed on through them. By incorporating Palestinians’ expertise, he thus believed to become closest to tapping into the knowledge of his own people's ancestors.^107^

The exclusion of native Palestinians and their knowledge about Palestine's plant world from the botanical garden further situated this project in a Western scientific tradition, which was typical for colonial botany. It signaled which type of expertise would be welcomed and taken seriously, which way of doing science and understanding the plant world would be given legitimacy and, in contrast, which ways of holding and communicating knowledge about plants was being sidelined. In this way, the Jerusalem botanists set themselves and their garden up as members of the Western scientific canon.

Importantly, the work done at the garden and by the Jerusalem botanists did have practical influence on the future of the Zionist movement in Mandatory Palestine. For example, in 1937, Eig was asked to testify in front of the Peel Commission on the question whether Palestine could sustain more people (a crucial aspect to enable increased Jewish immigration) and to prepare a map that could support the Zionist claims to Palestine internationally.^108^ Moreover, the scientists involved in the early stages of the department's work carried out research that is considered today the foundation of Israel's institutionalized study of botany. The Department of Botany thus fulfilled an important function in gathering several botanists and giving them the space and resources to carry out their research. For example, Eig's research on the flora of Palestine was developed during this time and, after his death, built upon and published by his colleagues there. The *Flora Palaestina* was the first systematic listing of the plants of Palestine in this scientific format.^109^

In sum, apart from the scientific functions the garden had as a part of the Hebrew University, the botanists imbued the garden with various political‐ideological tasks supporting Zionist goals of self‐determination and setting boundaries through the choice of which plants and knowledge to include. However, due to the very nature of the scientific field of botany, which can only thrive through international cooperation, the self‐sufficiency claimed by the Jewish botanists had to be quite porous. The botanical garden was thus intended to signal Jewish nationalist intentions, however, in its day‐to‐day operations, it was dependent on cross‐border cooperation and exchange.

## 4 Conclusion

In early twentieth‐century Ottoman and Mandatory Palestine, plant research carried out by Jewish scientists had various colonial connections. The Palestinian wheat that agronomist Aaron Aaronsohn made his flagship plant steered him towards fraternization with U.S. dryland farming and research, culminating in financial support for the first institutionalized Jewish plant research facility in Palestine, Aaronsohn's experiment station in Atlit along the Palestinian coast. The Jerusalem botanical garden, whose establishment botanists Otto Warburg and Alexander Eig promoted and spearheaded, was intended to showcase the independence of Jewish botanical expertise and self‐sufficiency within a broader network of gardens in the region into which the Jewish botanists would not just fit their garden but, from the outset, make claims of their own scientific superiority.

Both Palestinian wheat and the botanical garden influenced the scientific and political context in which plant research unfolded: wheat played an important role in the inception of plant research as carried out by Aaronsohn. Wheat here symbolizes a claim to nativeness, the search for origins of plants and their people, and potential for productivity and sustenance. In the case of the Jerusalem botanical garden, individual plants and their significance receded into the background as a more encompassing way of ordering nature was envisioned through the arrangement of plants in space. The botanical garden, in itself a deeply colonial construct, aimed at ordering nature into European scientific categories, thereby making knowledge claims over a space that, only a little while prior, had been foreign to most of the Jewish actors involved. The garden was intended to establish supposedly natural boundaries along both Jewish expertise and the Jewish nation in the land of Palestine. It shows how nature can be intended as a staging ground for the political goal of Zionist identity and nation building.

In both cases, the plant researchers involved tried to imbue the plant world around them with agency that they planned to work towards their goals of settling Palestine and creating a Jewish state. These attempts at fashioning companion species had quite different results. In the case of Palestinian wheat, the plant in the end impacted Zionism more than Zionism impacted the plant. However, research on Palestinian wheat is still ongoing and it remains to be seen whether Aaronsohn's promotion of Palestinian wheat will result in more long‐lasting consequences for this species, over a century after it was first brought into discussion. In the case of Hebrew University's botanical garden, the reverse is true: Zionism had more impact on the garden than the garden on Zionism. The reason the garden was created at all was, above all, an ideological one. Moreover, after the creation of Israel in 1948 and the abandonment of the garden due to its occupation by Jordan, it seems that its purpose had been fulfilled. When a new botanical garden was established in Jerusalem in 1985, it followed the more typical arrangement by modern botanical gardens of their plants into vegetation zones, including plants from all over the world rather than just from Israel.

## Conflict of interest

I have no conflicts of interest to disclose.

## Footnotes

1
Crosby 1972, 2004; Cronon 1983.

2
Bennett and Hodge 2011; Beattie et al. 2014; Kirchberger and Bennett 2020; Miller and Lindner 2025.

3
Ross 2017.

4
Griffiths and Robin 1997.

5
Brockway 1979; Drayton 2000; Blais 2023.

6
Cohen 1993; Braverman 2009; Tal 2013; Piekarska‐Baronet 2026.

7
Sufian 2007.

8
Cohen 2024.

9
Novick 2023.

10
Gilad 2021.

11
Karlinsky 2005.

12
von Suffrin 2019; Penslar 1991; Cohen 2024; Tesdell 2017; Novick 2023.

13 See, for example, Shafir 1996; Troen 2003; Aaronsohn 1995; Giladi 1975; Tal 2002.

14
von Suffrin 2019; Leimkugel 2005.

15
Haraway 2003.

16 Ibid., on 15.

17 Ibid., on 16.

18 Ibid., on 15.

19
Lorimer 2010; Tsing 2012; Beck 2021.

20
Klemun 2022.

21
Tsing 2012, on 145.

22 Ibid., on 148.

23 See Luregn Lenggenhager's article in this special issue.

24
Schiebinger and Swan 2005, on 2.

25
Kaiser 2015.

26 This work made Aaronsohn's and his associates’ political activism a popular topic in historiographical and popular scientific writing. See, for example, Engle 1959; Verrier 1995; Florence 2007; Katz 2019.

27
Katz 2001; Tesdell 2017; von Suffrin 2019.

28
Kraemer et al. 2022.

29
Katz 2001, on 7–8.

30
von Suffrin 2019, Chapter 5.

31 The most thorough and recent exceptions being Paz 2005 and Mörike 2025.

32
Tesdell 2017.

33
Katz 2001.

34 See, for example, von Suffrin 2019; Kraemer et al. 2022; Lack and van Slageren 2020; Mörike 2025. The use of the term discovery is by far the most widespread in the literature.

35
Tesdell 2017.

36
Katz 2001; Nevo 2014; Mörike 2025.

37 See, for example, Schiebinger 2007; Endersby 2008; Klemun 2006; Musgrave 2010.

38 This point has been made by Palestinian scholar Omar Tesdell who has argued that Aaronsohn appropriated Palestinian wheat through three different ways, namely taxonomic, agro‐climatic, and genetic practices. See Tesdell 2017.

39
Davis 2007; Bieling 2022a.

40
Tesdell 2017, on 46, 47.

41
Schiebinger 2007, on 194–225.

42
Tesdell 2017, on 46.

43 Ibid., on 46.

44
Spary 2005.

45 Another study uses the term progenitor for Palestinian wheat. See Kraemer et al. 2022.

46 By that time, Aaronsohn had built up quite the reputation through his research agenda on wheat in the U.S. See von Suffrin 2019, on 159

47
Fairchild 1938, on 369.

48
Brockway 1979; Kaiser 2021.

49
Seitz 2021; Koch 2022.

50
Blanckenhorn 1918.

51
Hargreaves 1977; Hargreaves 1993; Moon 2008.

52 Davidson and Kohler, on 209. It is not clear whether Aaronsohn presented his ideas to Roosevelt during his first visit to the U.S. and while Roosevelt was president, or later.

53
Aaronsohn 1906.

54
Aaronsohn 1910.

55
Tesdell 2017.

56 Ibid.

57
Katz 2001, on 14.

58 The board members read like a who is who of Jewish Americans: Julius Rosenwald (president), Morris Loeb (vice‐president), Paul M. Warburg (treasurer), Henrietta Szold (secretary), as well as Julian W. Mack, Jacob H. Schiff, Cyrus Adler, Louis Marshall, and Judah L. Magnes as further founders. See Davidson and Kohler 1928, on 203.

59
von Suffrin 2019, on 202; Birnhack 2024.

60
Schölch 1981; Karlinsky 2005.

61
von Suffrin 2019, on 138.

62
Katz 2001.

63
Tesdell 2017, on 47.

64
Blanckenhorn 1918, on 28.

65
Leimkugel 2005, on 137, 150.

66
Mörike 2025, on 98.

67
von Suffrin 2019, on 174.

68
Fairchild 1938, on 369.

69
von Suffrin 2019, on 176.

70
Penslar 1991; von Suffrin 2019.

71
Leimkugel 2005, on 128–152. In the 1930s, the experiment station moved to Rehovot and became part of the Hebrew University. It still exists today as “The Agricultural Research Organization—Volcani Institute” and is an independent affiliate unit of the Israeli Ministry of Agriculture and Rural Development.

72 For a more thorough overview over the different stages of the institutionalization of botany in Mandate Palestine, see Leimkugel 2005.

73 Works on the early years of the Hebrew University and its importance for the Zionist movement include: Lavsky 2005; Troen 1992; Cohen 2007; Katz and Heyd 1997; Macleod 2008.

74 The most recent and thorough exception being Mörike 2025.

75 His legacy was built upon by his colleagues Michael Zohary and Naomi Feinbrun‐Dotan whose research was crucial for the development of institutionalized botany in Israel after its creation in 1948.

76 Seminal on this Brockway 1979; Drayton 2000; McCracken 1997; Endersby 2008.

77
Headrick 1981.

78
Gänger 2020; Deb Roy 2017.

79 See correspondence at Hebrew University Archive (henceforth HUA), Jerusalem, for example fols. “Botanical Garden 1925–1930” and “Botanical Garden 1927–1930.”

80
von Suffrin 2021, on 187.

81 Ibid. For the conceptualization of the so‐called “New Jew” or Sabra, see Almog 2000.

82 Otto Warburg, Notes concerning the suitability of Lamport's land for a botanical garden, 28 March 1928, HUA, Jerusalem, fol. “Botanical Garden 1925–1930.”

83 Judah Magnes, Notes on Meeting with Pudsey, 16 December 1929, HUA, Jerusalem, fol. “Botanical Garden 1925–1930.”

84 Alexander Eig to Judah Magnes, 28 July 1929, HUA, Jerusalem, fol. “Botanical Garden 1925–1930.”

85 This is at least questionable. Semih Celik has described the first Ottoman Natural History Museum containing a garden, herbarium and glass houses and being scientifically oriented already in the 1830s. See Celik 2020.

86 Alexander Eig to Judah Magnes, 28 July 1929, HUA, Jerusalem, fol. “Botanical Garden 1925–1930.”

87 See, for example, Solomon Lamport to the Jewish National Fund, 27 December 1928, HUA, Jerusalem, fol. “Botanical Garden 1925–1930”; Otto Warburg, Opinion in the matter of appropriation of Mr. Lamport's property for a botanical garden, 13 March 1928, HUA, Jerusalem, fol. “Botanical Garden 1925–1930”; Otto Warburg, Notes concerning the suitability of Lamport's land for a botanical garden, 28 March 1928, HUA, Jerusalem, fol. “Botanical Garden 1925–1930.”

88 See, for example, Diagre 2011.

89
Novick 2023.

90
Bieling 2024.

91
Rakow and Lee 2015.

92
Klemun 2022.

93 Alexander Eig to Judah Magnes, 28 July 1929, HUA, Jerusalem, fol. “Botanical Garden 1925–1930.”

94 Ibid.

95 For a more detailed analysis of this letter, see Bieling 2022b.

96 Alexander Eig to Judah Magnes, 28 July 1929, HUA, Jerusalem, fol. “Botanical Garden 1925–1930.”

97
Kaiser 2021; Kaiser 2022; McCormack 2020.

98 See the Annual Reports of the Department of Agriculture and Fisheries, The National Library of Israel, Jerusalem, 2=PB 21, for example for the years 1931, 1932, or 1935.

99 M. T. Dawe et al., Report for the year 1935, Annual Reports of the Department of Agriculture and Fisheries, The National Library of Israel, Jerusalem, 2=PB 21.

100 F. J. Tear et al., Reports for the years 1931 & 1932, Annual Reports of the Department of Agriculture and Fisheries, The National Library of Israel, Jerusalem, 2=PB 21.

101 For Eig, see Mörike 2025, on 96–99.

102 Naomi Feinbrun, Curriculum Vitae, HUA, Jerusalem, fol. “Naomi Feinbrun‐Dotan I.”

103 For more detail on Hareuveni's conception of biblical botany, see Alon‐Mozes 2013, on 4.

104 Ephraim Hareuveni, “My Life History,” HUA, Jerusalem, fol. “Ephraim Hareuveni 1923–1930.”

105 Judah Magnes to J. N. Epstein, n.d., HUA, Jerusalem, fol. “Ephraim Hareuveni 1923–1930”; Otto Warburg to the Hebrew University, 18 March (?) 1927, HUA, Jerusalem, fol. “Ephraim Hareuveni 1923–1930.”

106
Mörike 2025, on 93–95.

107
Oren 2014, on 79.

108 Biography of Alexander Eig by the Alexander Silberman Institute of Life Science at Hebrew University Jerusalem, https://www.bio.huji.ac.il/en/content/eig‐alexander (accessed 5 March 2025). The Peel Commission was a British commission of inquiry sent to Palestine in 1936 to investigate the sources of conflict there. Its final report first suggested partition of the territory.

109 On Eig's scientific research and accomplishments, see Mörike 2025, on 95–99 and 105–109.
